# Operationalizing goal setting as an outcome measure in trials involving patients with frailty, multimorbidity or complexity

**DOI:** 10.1016/j.conctc.2024.101411

**Published:** 2024-12-07

**Authors:** Emma Tenison, Katherine Lloyd, Yoav Ben-Shlomo, Emily J. Henderson

**Affiliations:** a Ageing and Movement Research Group, Department of Population Health Sciences, Bristol Medical School, University of Bristol, Bristol, United Kingdom; b Older People's Unit, Royal United Hospitals Bath NHS Foundation Trust, Combe Park, Bath, United Kingdom; c The National Institute for Health and Care Research Applied Research Collaboration West (NIHR ARC West) at University Hospitals Bristol and Weston NHS Foundation Trust, United Kingdom

**Keywords:** Clinical trials, Goal attainment, Outcome measures, Complex interventions, Parkinson's disease, Frailty, Multimorbidity

## Abstract

**Background/aims:**

In the absence of disease-modifying therapies for Parkinson's disease, much research focuses on improving quality of life, health and wellbeing. It is important to evaluate potential treatments and innovative care models in a robust and standardised way. Disease-specific outcomes have limitations in older people, those with cognitive impairment, multimorbidity, disability or short life expectancy. We aimed to select, and adapt as needed, a primary outcome to evaluate a multicomponent intervention for people with parkinsonism.

**Methods:**

The multicomponent Proactive and Integrated Management and Empowerment (PRIME) model of care is being evaluated in the UK within a randomized controlled trial (RCT). We needed a meaningful outcome measure which could capture effects across multiple symptoms and domains; be suitable across the spectrum of disease stage/phenotype, including for participants with multimorbidity and/or cognitive impairment.

**Results:**

We have chosen the Bangor Goal-setting Interview and adapted it for use within the PRIME-UK RCT. This includes 4 steps: participants 1) identify an area to work on; 2) describe a specific goal; 3) rate current attainment, readiness to change and goal importance; and 4) attainment is followed up 3-monthly. Change in ratings across three to five individualised goals on a standardised scale can be compared between trial arms.

**Conclusion:**

We demonstrate how a goal-orientated outcome can be operationalized within a complex intervention trial for parkinsonism. Parkinsonism is an exemplar multisystem, heterogeneous condition, predominantly affecting older people. There is scope to use goal-orientated outcome measures more widely in trials involving patients living with frailty, multimorbidity and/or clinical complexity.

## 1 Background and aims

In the absence of disease-modifying therapies for Parkinson's disease (PD), much research focuses on improving quality of life, health, and wellbeing. A new care model, Proactive and Integrated Management and Empowerment in Parkinson's (PRIME) [1], is a multicomponent intervention being evaluated within a randomized controlled trial (RCT) which is enrolling patients with parkinsonism, and therefore includes those with frailty, multimorbidity and/or cognitive impairment as well as informal caregivers [2]. This intervention aims to improve the experience of living with PD and align care with ‘what matters most’ to individuals [3]. We sought to assess benefit of this innovative care model using an outcome which is aligned to the project aims, captures the hypothesised impact and is meaningful to patients.

Patient-reported outcome measures (PROMs), which collect outcomes directly from the people who experience them [4] are increasingly being used alongside or instead of biomarkers and measures of morbidity, mortality and healthcare use [5]. Many of these are disease-specific which has limitations, particularly in older people, as well as those with multiple long-term conditions, disability or short life expectancy in whom there are often trade-offs when considering multiple interventions/treatments [6].

Given the complexity and heterogeneity of PD, PROMs focusing on a single domain, such as motor symptoms, are often not appropriate and risk overlooking the spectrum of experience. Conventional outcome measures may not be validated for proxy completion on behalf of someone lacking capacity [7] and, whilst some outcomes have been endorsed for use in PD dementia [8], floor and ceiling effects may render them unsuitable for use in individuals with intact cognition, precluding their use in a trial recruiting across the disease spectrum. When considering a complex intervention like the PRIME-UK care model, there is a need for PROMs which capture the downstream effects of the multiple components, which are aiming to produce impact across physical, psychological and social domains.

Given the limitations of traditional PROMs, novel outcomes that can more holistically capture personal experiences and wellbeing have been proposed. Cools *et al*. suggested happiness as an outcome in PD studies [9] but this also has limitations since wellbeing is a multi-dimensional construct encompassing psychological wellbeing, not simply the pursuit of happiness [10]. Traditional outcomes can be made more patient-centred by reframing them into goal-orientated outcomes: instead of using a pain inventory for arthritis, we may assess pain control sufficient to carry out an activity important to the patient [6]. Instead of disease-free survival, the goal-orientated outcome would be survival until a personal milestone, or there may be no survival outcome if this were not a patient priority [6]. Goal attainment scaling (GAS) [11] requires individuals to set meaningful goals with progress subsequently rated on a standardised scale and a formula applied to calculate an aggregated score [11]. In addition to clinical use as an intervention to motivate behavioural change, goal-orientated approaches have been used to evaluate interventions in research settings, including amongst people with multiple sclerosis [12], older adults living with frailty [13] and PD patients [[14], [15], [16], [17], [18]]. We aimed to select a suitable primary outcome measure and adapt it for use within the PRIME-UK RCT.

## 2 Methods

### 2.1 Selection of a primary outcome measure

The PRIME RCT, approved by the London-Harrow Research Ethics Committee (REC reference 21/LO/0387), aimed to enrol a ‘real-world’ population to make the findings generalisable to clinical practice. The outcome measure therefore needed to be suitable for use in participants across the spectrum of disease stage and phenotype, including those with cognitive impairment and multimorbidity, as well as meaningful to patients and able to capture the effect of a complex intervention acting across multiple domains.

Goal-orientated outcomes offered a way to elicit an individual's goals, and generate a numerical score to allow assessment of attainment over time and comparison between patients with multiple personalised goals. From a literature review, we identified four goal-based measures which have been used to evaluate interventions, rather than goal-setting as an intervention itself, each of which we deemed to have pros and cons based on the practicality of use within a trial, prior use in PD and psychometric properties (Table 1). We selected the Bangor Goal-setting Interview (BGSI) as the PRIME RCT primary outcome measure because it was developed for research purposes, rather than for clinical settings with subsequent adaptation, and has advantages for this context (Table 1), including a freely-available manual and the flexibility to adapt it to specific settings [19]. It has also been shown to be feasible for use with people with PD dementia and Dementia with Lewy Bodies [20,21].

**Table 1:** Interventional studies, including trials, which included a goal-orientated outcome measure.

Goal-orientated outcome measure	Brief description of measure	Examples of uses including study population	Pros	Cons
Goal attainment scaling (GAS) [11]	Main problem area/domain is identified during an interview with the patient and goal(s) agreed jointly; the expected outcome defined as achievement level 0 is agreed and descriptors are pre-agreed for level −1/-2 and +1/+2, representing a worse or better outcome than expected respectively. Each goal is rated on the 5-point scale at follow up.	- A single blind RCT of cognitive training, transfer training and psychomotor training in people with PD and MCI [14] - A pilot, double-blind RCT of memantine versus placebo for PD dementia [15,29] - A service evaluation of dance classes for PD [30] - An RCT of comprehensive geriatric assessment as an adjunct to usual care in frail older patients [13]	- Goals can be weighted to account for the importance to the patient and/or anticipated difficulty - There is no requirement to record baseline scores (although these are usually rated as −1) - Performance is judged against predefined levels.	- Potentially time-consuming to define pre-determined levels for each of the 5 outcome score levels (−2 to +2) [11] - Zero and minus scores can be discouraging for patients [11] - If baseline score is recorded as −1 (which allows for deterioration to −2), it is not possible to record if a goal is partially achieved (but does not warrant the expected outcome level of 0) [11].
Patient-specific functional scale (PSFS) [24]	A self-reported measure in which patients list up to five activities they struggle to do because of their condition/problem. For each, they rate from 0 to 10 their ability to perform the activity at baseline and then subsequently at follow-up.	- A prospective, observational pre-post study of goal-orientated rehabilitation in individuals with a neurological condition (including PD and MS) - RCTs in populations with COPD [31], low back pain [32] and cervical radiculopathy [33].	- May be less time-consuming and require fewer resources than a semi-structured interview as the process is patient-directed - Reported to have good reliability, validity and responsiveness in a range of musculoskeletal conditions including mechanical back pain [24].	- Does not involve a semi-structured interview so the patient is not guided/supported through the process which may be challenging for some patient groups including those with cognitive impairment - Lack of evidence for use in neurological disease [24].
Bangor Goal-setting interview (BGSI) [19]	The patient sets goals in a collaborative interview with the option for an ‘informant’ to provide input. Goal attainment descriptors are pre-defined to indicate what would constitute e.g. 0, 50, 100 % attainment. Current attainment is rated on a 1 to 10 scale, with option to rate importance and motivation to change. Attainment is rated from 1 to 10 at follow-up.	- Primary outcome in a single blind pilot RCT of cognitive rehabilitation versus relaxation therapy for PD dementia/Lewy Body Dementia [16] - Primary outcome in a single-blind RCT of goal-orientated cognitive rehabilitation in early-stage dementia, the’GREAT’ trial [34]	- Developed for research purposes [35] and the manual includes specific guidance on use of BGSI in an RCT [19] - The manual and recording sheet are freely available for researchers to use and include resources to guide the interview, written in an accessible format, suitable for individuals with cognitive impairment - Incorporates the option, not only for an informant to support the process, but also for a caregiver to independently rate attainment - The structure allows some flexibility to adapt the measure for use in different settings - Descriptors set at baseline can help with subsequent rating of attainment	- Potentially time-consuming to define the descriptors at baseline - Limited information on the psychometric properties
Canadian Occupational Performance Measure (COPM) [28]	Semi-structured interview in which the patient is guided to consider 3 areas: productivity, self-care and leisure. Patients choose up to 5 problems and rate current performance and satisfaction on a scale of 1–10, where a higher score represents greater performance or satisfaction. Performance and satisfaction are rated again at follow-up.	- A case series (pre and post testing) of an 8-week treatment program to train for the use of compensatory external aids to achieve personalised goals [17]	- Evidence supports the reliability, construct validity and responsiveness [28].	- Specification of the 3 areas (productivity, self-care and leisure) may not be relevant to all individuals or interventions - The measure and related resources must be purchased

GAS: goal attainment scaling; RCT: randomized controlled trial; PD: Parkinson's Disease; PSFS: patient-specific functional scale; MS: Multiple Sclerosis; BGSI: Bangor Goal-setting Interview; COPM: Canadian Occupational Performance Measure.

We adapted the BGSI manual processes [19] for use in our trial. Our sample size calculation was based upon a minimal clinically important difference (MCID) of 2 points on the BGSI, as used in the GREAT trial [22], based on recommendations for the Canadian Occupational Performance Measure (COPM), a similar rating scale (personal communication, Professor Linda Clare, October 3, 2020).

## 3 Operationalizing goals as an outcome measure

3.1 Use of the Bangor Goal-setting Interview within the PRIME-RCT Agreement of goals before randomization avoids prior knowledge of intervention status biasing goal selection [23]. During a collaborative interview the participant sets three to five goals with a trained researcher, with input from a family member/caregiver (an ‘informant’), particularly for participants with cognitive impairment [19]. Unlike the original BGSI, we do not pre-specify domains within which participants must identify goals since these are expected to be highly personalised.

**Fig. 1:**
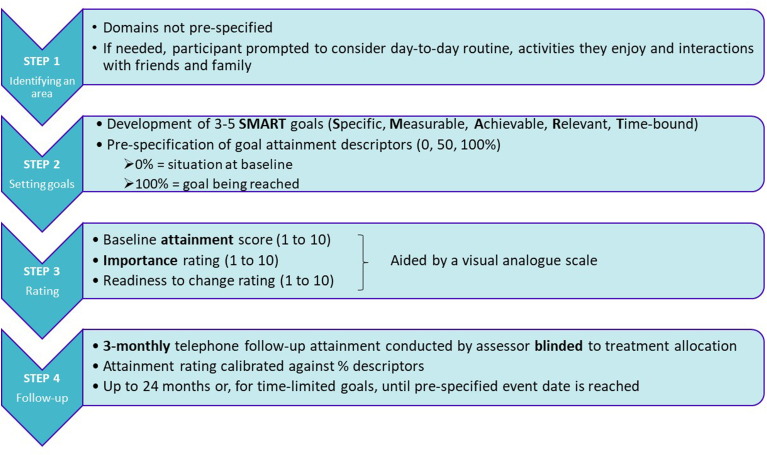
The procedure for using the Bangor Goal-Setting Interview within the PRIME randomized controlled trial prior to randomization.

After goal selection, percentage descriptors are specified to indicate what would constitute, for example, 50 % goal attainment. The participant is then asked to rate their current attainment, perceived importance and readiness to change numerically for each goal, with an option for an enrolled caregiver to independently rate attainment. The four steps are summarised in Fig. 1, described in more detail below and illustrated in Table 2 with an example.

**Table 2:** Applying the adapted Bangor Goal-setting interview to set and follow up a goal within the PRIME randomised controlled trial, where text in italics indicates how the researcher would guide the participant through the process.

**STEP 1**	
Specify the area (domains not pre-specified^a^) *“Parkinson's can impact on daily life in various ways. There are different things that people may want to change to make their lives more enjoyable. We are asking everyone taking part in this trial to come up with several goals that they would like to achieve. A goal might be something that you currently find difficult to do and wish you could do more easily, or without getting frustrated. Or a goal might be something that you are currently not doing and would like to do more of. Or it might be something that you would like to learn how to do.”*	Going to the gym
**STEP 2**	
SMART goal statement (100 % attainment)	I will go to the gym to exercise 3 times per week for 45–60 min
Description of current attainment (0 % attainment)	I have membership but do not go at all
Goal attainment descriptor^b^ (50 %)	I will go 1–2 times per week or 3 times for a shorter session (e.g. 20 min s)
**STEP 3**	
Rating of attainment	1
Rating of importance^b^	7
Rating of readiness to change^b^	6
**STEP 4** (e**ach** 3 monthly follow up conducted by blinded assessor^b^)	
Free text record of current activity in relation to goal *“Last time we spoke about going to the gym. Your goal was to go to the gym 3 times* per *week to exercise for 45–60 min. How are things going with that?”*	“Due to a recent small fracture of my right ankle I had to wear a boot and rest for 6 weeks, but I'm gradually starting to exercise again. In the last month, I have gone to the gym 4 times for 30–40 min”
Participant raw attainment score (without reminder of prior rating in step 3^b^) *“What score would you give yourself on a scale of 1-10 for how well you are doing now? 10 would be that you are doing it very successfully, 1 would be that you are not doing it at all or not successfully at all."*	3
Calibrated attainments scores^a^ *“At your baseline visit, you said you had membership but were not going to the gym and you gave yourself an attainment score of 1. We decided at that time that achieving your goal by 50 % or making it half way to your goal would be going to the gym 1–2 times* per *week or going 3 times for shorter sessions (*e.g. *20 min). Based on what you have just told me, it sounds like you are more than 50 % of the way to achieving your goal. I wonder whether the new score should be somewhere around 6- would you agree with this?"*	Interviewer-decided attainment score calibrated from baseline attainment score using % descriptors6
	Calibrated score as decided by participant following discussion5

SMART: Specific, Measurable, Achievable, Relevant, Time-bound.

a Indicates an aspect of the process which is specific to the operationalisation of BGSI within PRIME-RCT.

b Indicates an aspect of the process which was specified as an option within the BGSI manual and which was incorporated into the PRIME-RCT.

### 3.1 Follow-up of attainment

At follow-up, the participant describes their current activity in relation to each goal. They then rate their attainment numerically, without reminder of their prior rating. The researcher uses the percentage descriptors defined at baseline to decide on a researcher-determined calibrated attainment score which reflects the description of current attainment. In dialogue with the participant, the researcher assists them to decide on the participant-determined calibrated score.

Sometimes goals cannot be attained due to factors beyond the participant's control e.g. a dance class not attended because of cancellation; giving a low attainment score would not reflect participant wellbeing, so the goal rating is omitted, and an explanation recorded. However, to avoid overestimation of goal attainment, low attainment is recorded if the reason relates to patient wellbeing; for example, they did not attend the dance class due to an injury. This distinction is an adaptation to the BGSI.

### 3.2 Frequency of data collection

A key consideration was the frequency of goal follow-up, acknowledging that participants may select goals which are achievable in different timeframes and may subsequently wish to add another goal to replace an event-related goal. In the PRIME RCT, participants specify at baseline the timeframe in which they intend to achieve each goal. Regardless, all goals are followed up 3-monthly by a blinded assessor. Participants are reminded not to reveal their treatment allocation during the call and any unblinding is recorded.

### 3.3 Derivation of the score

**Fig. 2:**
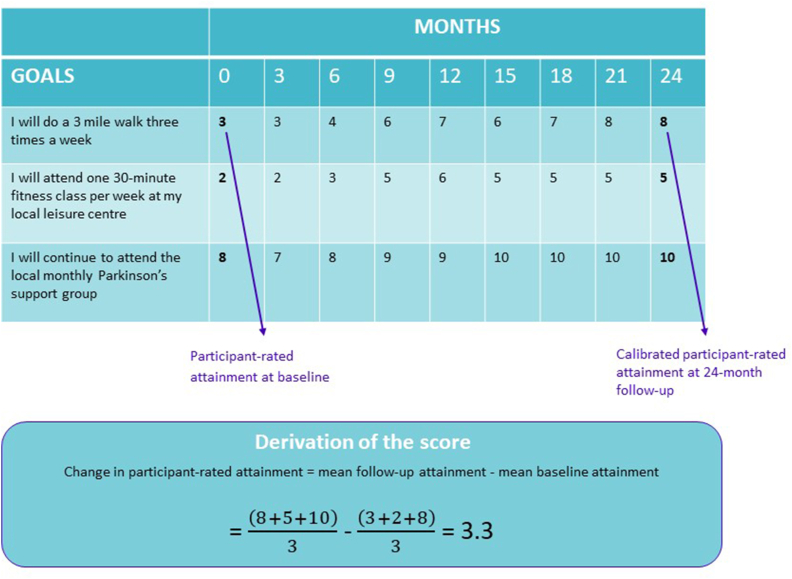
A worked example showing the process for calculating the change in attainment for one participant who had set three goals.

The primary outcome is change in participant-rated attainment, calculated as shown in Fig. 2. Whereas for classical measurement instruments a cut-off score may indicate a particular diagnosis and individual scores can be compared to normative data, intervention evaluation using a goal-based outcome relies on comparison of two group means [23]. Follow-up at all 3-monthly timepoints will facilitate a secondary longitudinal analysis to determine whether any improved goal attainment is maintained. Secondary analyses will include change in caregiver-rated attainment and for the three goals rated as most important.

## 4 Conclusion

We have described how a goal-orientated outcome measure was adapted for use in a trial involving individuals with complexity, frailty and multimorbidity. As the trial progresses, we will gain crucial real-world experience of applying this in people with parkinsonism, including the time and resources required. We will also gather quantitative data on the numbers of goals set, typical domains chosen, agreement between patient and caregiver scores and the proportion unable to rate attainment (e.g. due to cognitive impairment).

The lack of a defined MCID for the BGSI is a limitation; determining this in a parkinsonism population would add to its utility and help to inform whether BGSI is better able to detect a meaningful difference than some other outcomes, as suggested [[24], [25], [26]]. Whilst the evidence for responsiveness is encouraging [27], evidence for the validity and reliability of goal-orientated outcome measures is limited and relates mainly to the COPM [28]. Future work could establish whether an improvement in goal attainment is associated with downstream benefits such as reduced unplanned hospitalisation, which would demonstrate predictive validity.

There are many potential benefits to wider use of goal-orientated outcome measures in clinical trials. They provide a holistic outcome not limited to specific conditions and with universal relevance, important for trials with broad inclusion criteria, enrolling heterogenous populations including adults with frailty and multimorbidity. Above all they aim to provide a meaningful measure of “what matters”. Knowledge gained from use of the adapted BGSI in the PRIME 10.13039/100014144RCT will help to support its implementation in conditions besides neurodegenerative disease, the focus of its use so far.

## CRediT authorship contribution statement

**Emma Tenison:** Writing – original draft, Methodology, Conceptualization. **Katherine Lloyd:** Writing – review & editing, Project administration, Methodology. **Yoav Ben-Shlomo:** Writing – review & editing, Supervision, Methodology, Conceptualization. **Emily J. Henderson:** Writing – review & editing, Supervision, Methodology, Funding acquisition, Conceptualization.

## Trial registration number

NCT05127057.

## Grant support

10.13039/501100000324Gatsby Charitable Foundation, GAT3676.

## Funding sources

The PRIME-UK programme is funded by the 10.13039/501100000324Gatsby Charitable Foundation (GAT3676).

## Declaration of competing interest

The authors declare the following financial interests/personal relationships which may be considered as potential competing interests: ET is funded by a 10.13039/501100000272National Institute for Health and Care Research Academic Clinical Lectureship and has received a speaker honorarium from the Neurology Academy.

KL is in receipt of PhD fellowship funding from The Gatsby Foundation.

EH is HEFCE funded by University of Bristol for her academic work and has received honoraria from the Neurology Academy and travel support from Bial.

YBS is partly funded by National Institute for Health and Care Research Applied Research Collaboration West (NIHR ARC West) and University of Bristol and has received funding from Parkinson's UK, Royal Osteoporosis Society, MRC, HQIP, Templeton Foundation, Versus Arthritis, Wellcome Trust, National Institute of Health Research, Gatsby Foundation.
